# Immunotherapeutic Potential of Mollusk Hemocyanins in Combination with Human Vaccine Adjuvants in Murine Models of Oral Cancer

**DOI:** 10.1155/2019/7076942

**Published:** 2019-01-20

**Authors:** Juan José Mora Román, Miguel Del Campo, Javiera Villar, Francesca Paolini, Gianfranca Curzio, Aldo Venuti, Lilian Jara, Jorge Ferreira, Paola Murgas, Alvaro Lladser, Augusto Manubens, María Inés Becker

**Affiliations:** ^1^Fundación Ciencia y Tecnología para el Desarrollo (FUCITED), Avenida Alcalde Eduardo Castillo Velasco 2902, Santiago, Chile; ^2^HPV-Unit-UOSD Tumor Immunology and Immunotherapy, IRCCS Regina Elena National Cancer Institute, Via Elio Chianesi 53, Rome, Italy; ^3^Human Genetics Program, Institute of Biomedical Sciences (ICBM), Faculty of Medicine, Universidad de Chile, Avenida Independencia 1027, Santiago, Chile; ^4^Clinical and Molecular Pharmacology Program, Institute of Biomedical Sciences (ICBM), Faculty of Medicine, Universidad de Chile, Avenida Independencia 1027, Santiago, Chile; ^5^Laboratory of Immunoncology, Fundación Ciencia & Vida, Av. Zañartu 1482, Santiago, Chile; ^6^BIOSONDA Corporation, Avenida Alcalde Eduardo Castillo Velasco 2902, Santiago, Chile

## Abstract

Mollusk hemocyanins have been used for decades in immunological and clinical applications as natural, nontoxic, nonpathogenic, and nonspecific immunostimulants for the treatment of superficial bladder cancer, as carriers/adjuvants of tumor-associated antigens in cancer vaccine development and as adjuvants to dendritic cell-based immunotherapy, because these glycoproteins induce a bias towards Th1 immunity. Here, we analyzed the preclinical therapeutic potential of the traditional keyhole limpet hemocyanin (KLH) and two new hemocyanins from *Concholepas concholepas* (CCH) and *Fissurella latimarginata* (FLH) in mouse models of oral squamous cell carcinoma. Due to the aggressiveness and deadly malignant potential of this cancer, the hemocyanins were applied in combination with adjuvants, such as alum, AddaVax, and QS-21, which have been shown to be safe and effective in human vaccines, to potentiate their antitumor activity. The immunogenic performance of the hemocyanins in combination with the adjuvants was compared, and the best formulation was evaluated for its antitumor effects in two murine models of oral cancer: MOC7 cells implanted in the flank (heterotopic) and bioluminescent AT-84 E7 Luc cells implanted in the floor of the mouth (orthotopic). The results demonstrated that the hemocyanins in combination with QS-21 showed the greatest immunogenicity, as reflected by a robust, specific humoral response predominantly characterized by IgG2a antibodies and a sustained cellular response manifesting as a delayed hypersensitivity reaction. The KLH- and FLH-QS-21 formulations showed reduced tumor development and greater overall survival. Hemocyanins, as opposed to QS-21, had no cytotoxic effect on either oral cancer cell line cultured *in vitro*, supporting the idea that the antitumor effects of hemocyanins are associated with their modulation of the immune response. Therefore, hemocyanin utilization would allow a lower QS-21 dosage to achieve therapeutic results. Overall, our study opens a new door to further investigation of the use of hemocyanins plus adjuvants for the development of immunotherapies against oral carcinoma.

## 1. Introduction

Hemocyanins, large respiratory glycoproteins found in certain mollusks, have a broad range of beneficial biomedical effects due to their potent Th1 adjuvant activity [1]. The several notable uses for hemocyanins include their presence as a key component in therapeutic vaccines for cancer due to their useful carrier qualities [2, 3]. Hemocyanins are also used as nonspecific immunostimulants for the treatment of superficial bladder cancer (SBC), for which these glycoproteins have demonstrated several advantages over the standard immunotherapeutic procedure involving the use of *Bacillus* Calmette-Guérin, primarily because hemocyanins are molecules instead of living organisms [4]. The hemocyanin known as keyhole limpet hemocyanin (KLH), from the gastropod *Megathura crenulata*, has been used for decades in immunological and clinical applications for the abovementioned purposes [5–7].

To date, KLH has been applied in various *in vitro* and preclinical studies to determine its effectiveness against other cancers, such as Barrett's adenocarcinoma [8]; pancreatic, breast, and prostate cancer [9]; and melanoma [10, 11]. However, its production relies fully on a single natural source because the recombinant production of hemocyanin has been unsuccessful to date; this limited availability has prompted significant interest in studying other hemocyanins. Thus, gastropod hemocyanins from *Concholepas concholepas* (CCH) [12], *Fissurella latimarginata* (FLH) [13], *Haliotis tuberculata* [14], *Helix pomatia* [15], and *Rapana venosa* [16, 17], which have been extensively characterized in terms of their biochemical properties and immunomodulatory/adjuvant effects, have emerged as potential candidates to complement or substitute for KLH [1, 18].

The intrinsic immunogenicity and adjuvanticity of hemocyanins in mammals have been attributed to their xenogeneic character, immense size (approximately 4 to 8 MDa), and intricate quaternary structure. Mollusk hemocyanin molecules have a cylindrical form composed of 10 subunits associated in dimers, each subunit ranging in size from 350 to 550 kDa. This basic decamer structure can, in certain species, including those in this study, associate in pairs to form didecamers approximately 35 nm in diameter and 38 nm in height, which are easily observable via electron microscopy [19, 20]. Another notable feature fundamental to the structure of these glycoproteins is their carbohydrate content, which has been implicated in their antitumor effects in SBC [1, 5]. Hemocyanins possess mixtures of complex and heterogeneous glycans that reach up to 9% *w*/*w*, of which mannose is the most abundant carbohydrate [21, 22]. KLH has been further reported to contain the Thomsen-Friedenreich disaccharide antigen, or T antigen [23]. Thus, the immunotherapeutic effects of KLH in SBC are explained in part by a cross-reaction between antibodies that recognize glycotopes on the surfaces of tumor cells, triggering the effector functions of the immune response. This type of cross-reaction has also been reported between antibodies against hemocyanins from *Rapana thomasiana* and *Helix pomatia* and C-26 mouse colon carcinoma cells [24] and, similarly, between antibodies against CCH, FLH, and KLH and mouse and human melanoma cell lines [25]. In addition, the conformational stability of hemocyanins contributes to their adjuvant/immunostimulatory properties [26].

In the present study, we investigated the traditional KLH and two novel hemocyanins, CCH and FLH. We previously demonstrated that CCH and FLH have quaternary structures distinct from that of KLH. The KLH preparation comprises two independent isoforms that coexist, each composed of one type of subunit (KLH1 and KLH2) [27]. Although CCH also has two subunits (CCHA and CCHB), these subunits are intermingled in the molecule, forming heterodidecamers [12]. In contrast to these hemocyanins, FLH is composed of a single type of subunit that forms homodidecamers [13]. CCH and KLH showed similar immunogenic and immunotherapeutic properties in a murine bladder cancer model [28], and CCH, similar to KLH, has been demonstrated to be safe and useful as an adjuvant in dendritic cell- (DC-) based immunotherapy for patients with prostate cancer [29]. FLH was found to be more immunogenic and to exhibit more potent antitumor activity than CCH and KLH in a melanoma model [13].

We have focused our attention on a model of head and neck cancer, or oral carcinoma, because to date, there is no experimental evidence regarding the effects of hemocyanins in immunotherapy for this type of cancer. Human head and neck squamous cell carcinoma (HNSCC) is the sixth most common cancer worldwide and includes lesions in various anatomical sites, such as the lip, oral cavity, nose, sinuses, nasopharynx, oropharynx, hypopharynx, and larynx [30, 31]. This cancer is considered one of the most aggressive biologically malignant tumors, and long-term survival for patients is less than 50% [32]. Current therapeutic strategies for oral cancer treatment include surgery, radiation therapy, and chemotherapy, but these methods have shown limited efficacy in preventing both recurrence and tumor progression. Furthermore, a significant proportion of patients develop local invasion and metastasis [33, 34]. The prognosis of patients with this pathology is relatively poor, despite recent therapeutic advances [35]. It is therefore necessary to continue investigating novel therapeutic agents and strategies that can lead to the control and prevention of this disease.

Considering the aggressive and deadly malignant behavior of oral cancer, it is necessary to potentiate the antitumor activity of hemocyanins with adjuvants that have demonstrated efficacy in human vaccines [36–38]. Thus, we selected the following adjuvants: QS-21, a highly purified saponin extracted from the Chilean tree *Quillaja saponaria* Molina, which has proven to be among the most potent immunological adjuvants [39] and induces cytotoxic T lymphocytes along with the secretion of Th1-type cytokines, e.g., IL-2 and INF-*γ*, and IgG2a isotype antibodies [40–42]; AddaVax, an adjuvant related to the MF59 adjuvant, an oil-in-water formulation based on squalene that promotes a significant increase in antibody titers with balanced Th1/Th2 immune responses [37, 43]; and alum, which has been injected into billions of people [44] and whose mechanisms of action include the formation of a deposit, facilitating the release of antigen; the formation of a particle structure that promotes antigenic phagocytosis by antigen-presenting cells; and an increase in MHC-II expression and antigen presentation, directing the immune response towards a Th2-type response characterized by the presence of IgG1 isotype antibodies and IL-4 cytokine production [45–48].

In this context, the primary goals of this study were to compare the performance of CCH, FLH, and KLH in combination with alum, AddaVax, and QS-21 in terms of specific humoral and cellular immune responses. We then selected the best formulations, which were the hemocyanins combined with QS-21, to investigate their nonspecific antitumor effects in two murine models of oral cancer: MOC7 cells implanted in the superior flank (heterotopic model) [49] and bioluminescent AT-84 E7 Luc cells implanted in the oral pavement (orthotopic model), a novel model for HPV- (human papilloma virus-) related oral tumor [31], which allowed us to monitor tumor progression with an *in vivo* imaging system (IVIS). In this respect, approximately 20% of HNSCCs are HPV-positive [50], emphasizing the importance of hemocyanin treatment in combination with QS-21 in this orthotopic mouse model of oral cancer. Finally, as an approximation of the mechanism of action of the hemocyanin/QS-21 preparations, their effects on cell viability were evaluated *in vitro*.

## 2. Materials and Methods

### 2.1. Hemocyanins

The hemocyanins from *Concholepas concholepas* (Inmunocyanin®) and *Fissurella latimarginata* (*Blue-Vac*®), which were isolated under sterile and pyrogen-free conditions and suspended in PBS (0.1 M sodium phosphate, 0.15 M NaCl, pH 7.2), were provided by Biosonda S.A. (Santiago, Chile). KLH solution from *Megathura crenulata* was purchased through Merck (Darmstadt, Germany). The endotoxin contents of the hemocyanin preparations were determined using a PyroGene Recombinant Factor C Endotoxin Detection Assay Kit (Lonza Group, Walkersville, MD, USA). Chemical reagents were of professional analysis grade. Salt solutions were prepared with water for irrigation (Baxter Healthcare, Charlotte, NC, USA) and filtered through a 0.02 *μ*m membrane filter (Millipore, Billerica, MA, USA).

### 2.2. Adjuvants

Alum, in the form of an aluminum hydroxide wet gel suspension, was supplied by Sigma-Aldrich (Sigma-Aldrich, St. Louis, MO, USA); QS-21 was obtained from Desert King Chile S.A. (Quilpué, Región Valparaíso, Chile); and preclinical grade AddaVax was acquired from InvivoGen (San Diego, CA, USA). The formulations of hemocyanins with adjuvants were prepared aseptically immediately prior to administration to mice or cell cultures.

### 2.3. Experimental Animals

Female C57BL/6 mice (aged 8 to 16 weeks) were obtained from GrupoBios S.A. (Santiago, Chile) and the University of Chile. The mice were maintained in the animal care facility at the Biosonda Corporation with water and food *ad libitum*. This study was performed in accordance with the guiding principles for animal welfare of the National Commission for Science and Technology (CONICYT, Chile). C3H/He experiments were performed at the Regina Elena National Cancer Institute (Rome, Italy). Female mice (aged 8 to 16 weeks) were maintained under specific pathogen-free conditions at the Experimental Animal Department of the Regina Elena Institute. The animal experiments performed in this study were conducted according to institutional animal use guidelines and the Italian law (DL 116/92).

### 2.4. Cell Culture

The murine oral cancer cell line MOC7 (kindly provided by Dr. Ravindra Uppaluri, Department of Otolaryngology, Washington University School of Medicine, St. Louis, Missouri) was cultured according to Judd et al., with minor modifications [49]. The cells were cultured at 37°C in a 10% CO_2_ atmosphere in Dulbecco's medium (HyClone, Logan, UT, USA) containing 10% fetal bovine serum (FBS, HyClone), 100 IU/ml penicillin, and 100 mg/ml streptomycin (Gibco, Carlsbad, CA, USA). The murine squamous cell oral carcinoma cell line AT-84 E7 Luc was derived from parental AT-84 cells by transfection with HPV16 E7 and Luciferase genes. This cell line was cultured in RPMI medium (HyClone) supplemented with 10% FBS and antibiotics, as described by Paolini et al. [31]. Both cell lines were continuously propagated with successive transfers and harvested with trypsin/EDTA (Gibco).

### 2.5. Immunization Scheme and Anti-hemocyanin Humoral Immune Response Assessment by ELISA

Female C57BL/6 strain mice were separated into groups of 3 and immunized (day one) subcutaneously in the superior right flank with 50 *μ*g of each hemocyanin, either alone in 100 *μ*l of PBS or in combination with one of the adjuvants. For alum, 100 *μ*g of adjuvant was combined with 50 *μ*g of each hemocyanin in a total volume of 100 *μ*l of PBS. For AddaVax, 50 *μ*g of each hemocyanin was combined 1 : 1 (vol/vol) with adjuvant in a total volume of 100 *μ*l of PBS. For QS-21, 50 *μ*g of each hemocyanin alone was combined with 10 *μ*g of adjuvant in a total volume of 100 *μ*l of PBS. At day 16, a second immunization was performed via the same route with the same doses described above. At 37 days, serum samples were obtained. To measure the serum anti-hemocyanin antibody titers of the experimental animals, the procedure described by Oliva et al., was used [51]. Briefly, 96-well plates (Thermo Scientific, Rochester, NY, USA) were coated with 100 *μ*l/well of a solution containing 10 *μ*g/ml KLH, CCH, or FLH in PBS and incubated overnight at 4°C; then, the plates were blocked with 200 *μ*l of a 1% PBS-casein (Sigma-Aldrich) solution for one hour at 37°C. Sera were added, and serial twofold dilutions were made in 1% PBS-casein solution. The plates were incubated for two hours at 37°C, and the wells were then washed three times with 200 *μ*l of PBS-Tween 0.02%. Subsequently, 100 *μ*l of a 1% PBS-casein solution containing goat anti-mouse IgG (H + L) serum labeled with alkaline phosphatase (ALP; Thermo Scientific) was added at a 1 : 2500 dilution and incubated for 30 minutes at 37°C. The wells were washed and subsequently developed via a 20-minute incubation at 37°C with 100 *μ*l of a 1 mg/ml solution of pNpp (Merck) in ALP buffer. The reaction was stopped with 100 *μ*l of 3 N NaOH, and the optical density (OD) was read at 405 nm. As a control for the reaction specificity, the primary antibody was omitted. The results were expressed as the serum dilution versus OD. The antibody titer was defined as the serum dilution at which half of the maximal OD was reached.

### 2.6. Isotype Determination of Antibodies and Cytokine Determination by ELISA

For isotype determination, the procedure of Moltedo et al. [28] was used. Briefly, 96-well plates were coated with 100 *μ*l/well of a solution containing 10 *μ*g/ml KLH, CCH, or FLH in PBS and incubated with serial twofold dilutions of mouse sera as described above. Subsequently, 100 *μ*l of a 1% PBS-casein solution containing goat serum anti-mouse IgG subclasses (i.e., IgG1, IgG2a, IgG2b, and IgG3) conjugated to HRP (1 : 10,000 dilution) from Thermo Scientific was added and incubated for 30 minutes at 37°C. The plates were washed in the same manner as described above, and 100 *μ*l of 1-Step™ Ultra TMB-ELISA solution (Thermo Scientific) was added. The plates were incubated for 20 minutes at 37°C, the reaction was stopped with 100 *μ*l of 3 N H_2_SO_4_, and the OD was read at 450 nm. As a control for reaction specificity, primary antibodies were omitted. Anti-subclass antibody results are expressed as half of the maximal OD of appropriately diluted serum samples. Levels of the murine cytokines IFN-*γ* and IL-4 were measured in the sera of mice using commercial kits according to the manufacturer's instructions (BD OptEIA ELISA Set, BD Biosciences, San José, CA, USA).

### 2.7. Delayed-Type Hypersensitivity Reaction (DTH) to Evaluate T-Cell-Mediated Immune Responses

DTH determinations were performed according to Palacios et al. [25]. Briefly, footpad swelling induced by subcutaneous injection in the hind foot pad with 50 *μ*g of each hemocyanin dissolved in 20 *μ*l of PBS, or with 20 *μ*l of PBS or QS-21 (1 mg/ml) diluted 1 : 1 in PBS as controls, was measured in mice previously anesthetized intraperitoneally with ketamine (100 mg/kg). For these measurements, a high-precision digimatic caliper (Mitutoyo, Japan) was used, and measurements were made immediately prior to the injection of each hemocyanin and at 24, 48, and 72 hours post-challenge. The DTH reaction was plotted as the difference in thickness between the left and right feet over time.

### 2.8. Evaluation of the Immunotherapeutic Effects of Hemocyanins in Combination with QS-21 in an Oral Cancer Heterotopic Model

To measure the antitumor effects of hemocyanins in combination with QS-21, the general procedure of Moltedo et al. was used [28]. Groups of 3 mice were injected subcutaneously in the superior right flank with an initial sensitizing dose of either 100 *μ*g of KLH and FLH alone or 50 *μ*g of each of these hemocyanins in combination with 10 *μ*l of QS-21 in a total volume of 100 *μ*l of PBS. As controls, PBS and QS-21 were included. On the day designated 0, the mice were challenged in the same region with 1 × 10^5^ MOC7 cells injected into the superior right flank; after 7 days, the mice underwent intralesional immunotherapy, which was repeated five times after the priming doses at intervals of 3 days. The tumor incidence was evaluated by visual inspection and palpation. The tumor dimensions (length and width) were measured at intervals of 3–4 days up to day 23, prior to exponential growth of the tumor. Tumor volume was calculated according to the formula 0.52 × (length × width^2^). Mouse survival was measured over a period of 65 days. In another prophylactic bioassay, the mice (5/group) were primed and challenged in a manner similar to that described above, but immunotherapy was initiated on the day following the challenge with tumor cells and repeated five times at intervals of 3 days after the priming doses were administered. Tumor incidence was evaluated as described above up to day 30, after which the mice were euthanized for analysis of immune cell infiltration into tumor tissues, spleens, and lymph nodes, as described below.

### 2.9. Evaluation of the Immunotherapeutic Effects of Hemocyanin Formulations Plus QS-21 in an Oral Cancer Orthotopic Model

The general procedure described by Paolini et al., with modifications, was used [31]. At day −14, groups of 5 previously separated female C3H/He mice were injected in the superior right flank with either 100 *μ*g of KLH and FLH alone or 50 *μ*g of each of these hemocyanins in combination with 10 *μ*l of QS-21 in a total volume of 100 *μ*l of PBS. On day 0, the mice were anesthetized with Zoletil (tiletamine + zolazepam, Virbac, Milan, Italy) and injected with 100 *μ*l of a suspension of 6 × 10^5^ AT-84 E7 Luc cells in the floor of the mouth via an extraoral pathway. Next, treatment identical to priming was given at 24 hours and then at intervals of 3 days (i.e., days 4, 7, 10, and 14). Tumor growth was monitored with a Lumina IVIS® imaging system (Perkin Elmer, Waltham, MA, USA). Light emission was detected using the Lumina II IVIS® CCD camera system and analyzed with the Image-Pro® Premier software package. For image acquisition, the mice were anesthetized as described above. The mice were then injected intraperitoneally with 150 mg/kg D-luciferin (XenoLight D-luciferin, Keliper/PKI; Perkin Elmer). Ten minutes later, light emission was quantified in photons/second and visualized on a pseudocolor scale. The animals' exposure time was 5 minutes. The tumor area (pix^2^) was determined by drawing regions of interest (ROIs) on each location. In addition, the mice were bled on days 10 and 21 to obtain serum samples to determine titers against each hemocyanin as well as the antibody subclass. A DTH reaction against the tumors was performed on day 15 of the bioassay. Survival was assessed up to day 25 post-inoculation with the cells.

### 2.10. Analysis of Tumor-Infiltrating Immune Cells by Flow Cytometry

Groups of five C57BL/6 mice challenged with MOC7 cells under the immunotherapy protocol described above in the prophylactic bioassay were used to determine the presence of tumor-infiltrating lymphocytes and antigen-presenting cells (APCs), as described by Oyarce et al. [52] and Murgas et al. [53]. The mice were euthanized at day 30, and the tumors were removed. Single-cell suspensions of the tumors or organs were prepared using a solution containing collagenase IV (5 mg/ml, Gibco, Thermo Scientific) and DNase I (5 mg/ml, AppliChem, Maryland Heights, MO, USA) in RPMI supplemented with 0.5% FBS for 45 minutes at 37°C in a shaker bath. Then, the cells were stained with the following conjugated antibodies: anti-NK 1.1 Brilliant Violet 421 (PK136, BioLegend, San Diego, CA, USA), anti-CD-F4/80 PERCP (BM8, BioLegend), anti-CD11b APC (M1/70, BioLegend), anti-CD3 FITC (17A2, BioLegend), anti-CD4 PERCP (GK1.5, BioLegend), anti-CD8 APC/Cy7 (53-6.7, BioLegend), and Zombie Aqua (BioLegend) for 40 minutes at 4°C, then fixed with a 2% paraformaldehyde solution, and finally evaluated by flow cytometry (FACSCanto II, BD).

### 2.11. Cell Viability Assay

The alamarBlue® assay from Thermo Scientific was performed according to the manufacturer's instructions. Briefly, 2 × 10^4^ AT-84 E7 Luc tumor cells were seeded in 96-well plates. For the 72-hour trial, the cells were incubated with different concentrations of KLH, CCH, and FLH alone (0, 8, 16, 31, 63, 125, and 250 *μ*g/ml [8]) and with the adjuvant QS-21 (in the same proportions as the formulations described previously) three hours after seeding. At 72 hours, the medium was removed, and 100 *μ*l of alamarBlue reagent (prepared by dilution 1 : 10 in DMEM with 5% FBS) was added and incubated at 37°C. For the 24-hour trial, various formulations were applied 48 hours after seeding the cells; 24 hours later, the medium was removed, and 100 *μ*l of alamarBlue reagent prepared in a similar manner was added. The readings for both trials were performed 4 hours after alamarBlue application. The hemocyanins and QS-21 were used alone as controls; doxorubicin (10 *μ*M) was used as a positive control [54].

### 2.12. Transmission Electron Microscopy (TEM)

Cells were grown in 24-well tissue culture plates as described above, either with culture medium supplemented with the tested formulations or without the formulations as controls. Cells were collected by centrifugation at 1000 rpm for 10 minutes at room temperature and then fixed in 2% glutaraldehyde in 0.1 M cacodylate buffer, pH 7.4, for 24 hours, postfixed with 1% OsO_4_ (all reagents were from EMS, Hatfield, PA, USA), dehydrated in a graded series of acetone (Merck) and embedded in EMbed 812 (EMS), according to the method of Luft [55]. Thin sections (400-500 Å) were stained with 4% uranyl acetate (EMS) in methanol and lead citrate (EMS), according to Reynolds [56]. The preparations were imaged at 80 kV with a Philips Tecnai 12 electron microscope at the Servicio de Microscopía de la Pontificia Universidad Católica de Chile.

### 2.13. Statistical Analysis of the Results

Comparisons of humoral and cellular immune responses were analyzed using one-way ANOVA. IgG subclass comparisons were performed by two-way ANOVA. Comparisons between groups for antitumor activity were performed using an unpaired two-tailed Student's *t*-test. Survival curves were analyzed using the Kaplan-Meier method. All statistical analyses were conducted using GraphPad Prism 6.0 software (GraphPad Software, San Diego, CA, USA). *P* values less than 0.05 were considered statistically significant (^∗^*P* < 0.05, ^∗∗^*P* < 0.01, and ^∗∗∗^*P* < 0.001).

## 3. Results

### 3.1. Hemocyanins in Combination with QS-21 Adjuvant Showed a Stronger Th1 Response than Formulations with Alum or AddaVax Adjuvants

To induce the nonspecific immunotherapeutic effects of hemocyanins, priming with these glycoproteins is fundamental to harnessing their antitumor activity [28, 57]. For this reason, we first added different adjuvants (alum, AddaVax, and QS-21) to compare their enhancing effects on specific humoral and cellular immune responses against hemocyanins. C57BL/6 mice received two similar doses subcutaneously (at day 1 and day 16) of 50 *μ*g of each hemocyanin alone (KLH, CCH, and FLH) or in combination with one of the three adjuvants of interest. On day 37 after the secondary immunization, anti-hemocyanin titers and IgG subclasses were determined in the sera of the mice by indirect ELISAs.

**Figure 1:**
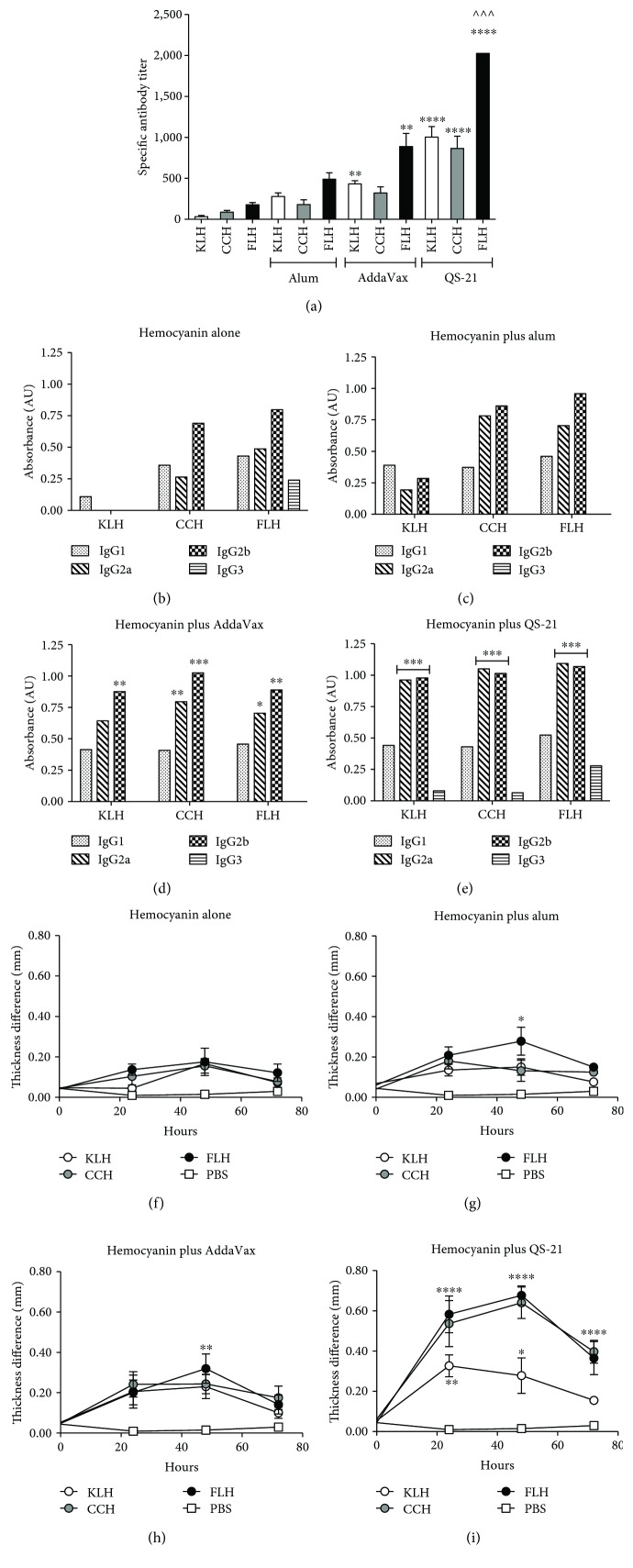
Specific humoral and cellular immune responses in C57BL/6 mice immunized with hemocyanins in combination with adjuvants. Groups of three female C57BL/6 mice were immunized subcutaneously on days 1 and 16 with one of the following treatments: 50 *μ*g of KLH, CCH, or FLH alone or in combination with 100 *μ*g of alum or 10 *μ*g of QS-21 in a 1 : 1 (vol/vol) ratio with AddaVax, all in a total volume of 100 *μ*l of PBS (vehicle). A serum sample was taken from each animal on day 37 after the second immunization for analysis by indirect ELISA. (a) Total anti-hemocyanin IgG titers in mouse sera. Values are presented as means ± SEM. The anti-hemocyanin IgG titers for each hemocyanin alone were compared by one-way ANOVA with the same hemocyanin in combination with an adjuvant. ^∗∗∗∗^*P* < 0.0001, ^∗∗^*P* < 0.01, and ^∧∧∧^*P* < 0.001. *n* = 2 independent experiments. (b) Anti-hemocyanin IgG subclass antibodies from a pool of mice immunized with each hemocyanin alone. For these comparisons, half of the maximum OD obtained for each of the isotypes with the different treatments was used [28]. Representative experiments in which the IgG1, IgG2a, IgG2b, and IgG3 titers from each of the treatments were compared by two-way ANOVA. ^∗∗∗^*P* < 0.001, ^∗∗^*P* < 0.01, and ^∗^*P* < 0.05; *n* = 2 independent experiments. (c) Mice immunized with hemocyanins in combination with alum. (d) Mice immunized with hemocyanins in combination with AddaVax. (e) Mice immunized with hemocyanins in combination with QS-21. (f) DTH footpad reactions induced by intradermal challenge with hemocyanins alone in mice immunized with each hemocyanin alone. The DTH test was performed by applying each hemocyanin treatment to the left feet of the mice and measuring the difference in thickness from the right foot at the start of the experiment (time 0) and after 24, 48, and 72 hours. (g) Mice immunized with hemocyanins in combination with alum. (h) Mice immunized with hemocyanins in combination with AddaVax. (i) Mice immunized with hemocyanins in combination with QS-21. Values are presented as means ± SEM. One-way ANOVA was used to compare the foot thickness of the control group at different measurement time points to those of the other treatments at the same time points. ^∗∗∗∗^*P* < 0.0001, ^∗∗∗^*P* < 0.001, and ^∗∗^*P* < 0.01, ^∗^*p* < 0.05; *n* = 2 independent experiments.

As shown in Figure 1(a), there were no significant differences between the titers against each hemocyanin alone (approximately 1 : 33 dilution for KLH, 1 : 90 for CCH, and 1 : 180 for FLH) and each hemocyanin in combination with alum (approximately 1 : 280 dilution for KLH, 1 : 180 for CCH, and 1 : 490 for FLH). Although alum can block Th1 responses [48], it was tested because it is currently the most widely used adjuvant for human and veterinary vaccines. Thus, the poor results obtained with alum are disappointing but somewhat expected, reinforcing current interest in the investigation of new adjuvants [58]. In the case of AddaVax, significant differences were observed with KLH and FLH (approximately 1 : 430 and 1 : 890 dilutions, respectively) in contrast to CCH (approximately 1 : 320 dilution). Although this result was encouraging, AddaVax cannot be easily administered. Indeed, AddaVax is an emulsion and must be used at a high dose (1 : 1 vol/vol) with respect to the other components in the formulation, making it a metastable system [59] requiring vigorous agitation after combination with hemocyanins prior to administration. The best results were obtained for these three hemocyanins in combination with the QS-21 adjuvant (approximately 1 : 1,000 dilution for KLH, 1 : 865 for CCH, and 1 : 2,025 for FLH). Furthermore, the administration of this formulation was easy: it was possible to obtain a homogeneous system with gentle agitation after combination with the hemocyanins. The Th1- and Th2-type profiles in the serum showed that the formulations consisting of hemocyanins alone and in combination with alum (Figures 1(b) and 1(c), respectively) yielded no significant differences between IgG isotypes. In contrast, hemocyanins with AddaVax (Figure 1(d)) and particularly those with QS-21 (Figure 1(e)) showed an IgG2a- and IgG2b-predominant response, confirming their strong bias towards a Th1-type response, as previously described [42, 43]. Additionally, the INF-*γ* and IL-4 cytokine levels were examined in the mouse sera to characterize the Th1 and Th2 immune profiles, respectively, for each formulation of hemocyanin and adjuvant. However, the results were negative, because the serum sampling times were long after the inoculation time (21 days after secondary immunization); consequently, the expected increase in IFN-*γ* to corroborate the results of the isotype analysis had already passed.

The cellular immune responses in the mice were then determined at day 50 of the bioassay by measuring the anti-hemocyanin DTH reaction. Fifty micrograms of each hemocyanin alone was used to treat a specific group by subcutaneous application to the left footpad of each animal; the right footpad was used as a control. Measurements were performed at the time of experiment initiation (time 0) and at 24, 48, and 72 hours post-injection of each hemocyanin. As shown in Figure 1(f), treatments with each mollusk hemocyanin alone showed a slight but not significant DTH reaction, which increased significantly when alum (Figure 1(g)) or AddaVax (Figure 1(h)) was combined with FLH at 48 hours post-challenge. Improved DTH reactions were observed for all hemocyanins in combination with QS-21 at 24 and 48 hours post-challenge (Figure 1(i)). This result suggests that QS-21 not only generates a greater humoral response than the other adjuvants, as discussed above, but also maintains a population of effector and memory T lymphocytes, as previously described [60]. Thus, QS-21 in combination with hemocyanins can induce rapid T-cell migration to the site of antigen application by 24 hours after administration and maintain this migration for 72 hours.

Collectively, these results are consistent with those of our previous studies demonstrating the superior performance of immunogens based on FLH [13, 25]. Thus, in the subsequent bioassays with mice, we decided to evaluate only FLH, and KLH was used as positive control. In addition, these data demonstrated that hemocyanins in combination with QS-21 induced robust humoral and cell-mediated immune responses compared to hemocyanins in combination with alum or AddaVax.

### 3.2. Hemocyanins in Combination with QS-21 Adjuvant Retard Tumor Progression in a Heterotopic Model of Oral Cancer

**Figure 2:**
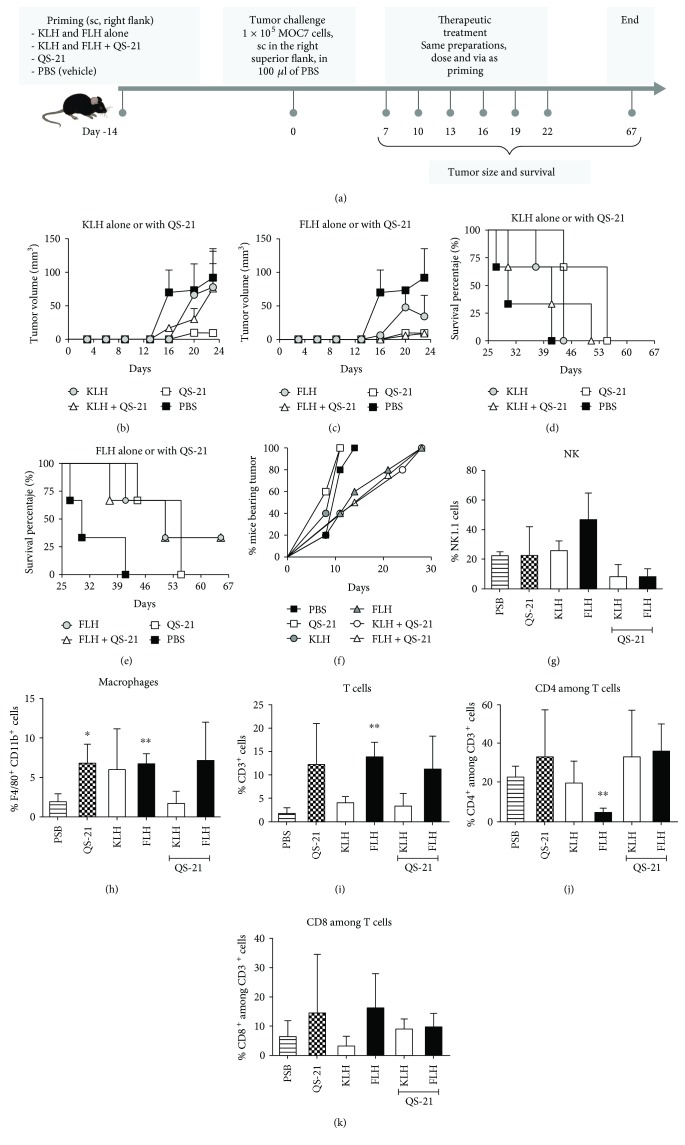
Antitumor effects of KLH and FLH in combination with QS-21 adjuvant in a heterotopic mouse model of oral cancer. (a) Experimental scheme. Three C57BL/6 mice were primed subcutaneously in the superior right flank (day −14) with the following treatments: 100 *μ*g of hemocyanin alone in 100 *μ*l of PBS (vehicle) or 50 *μ*g of each hemocyanin in combination with 10 *μ*g of QS-21 in a total volume of 100 *μ*l of PBS. Controls were 10 *μ*g of QS-21 alone in 100 *μ*l of PBS and 100 *μ*l of PBS. For the tumor cell challenge (0 days), the mice received 1 × 10^5^ MOC7 oral cancer cells subcutaneously in the superior right flank. After 7 days, the animals received an intralesional injection identical to the priming dose, and these treatments were then repeated 5 times at intervals of 3 days. (b) Effects of KLH alone or in combination with QS-21 on tumor growth. Data are shown as means ± SEM of one experiment with *n* = 3 mice per group; *P* not significant, two-tailed Student's *t*-test. (c) Effects of FLH alone or in combination with QS-21. (d) Effects of KLH alone or in combination with QS-21 on mouse survival. (e) Effects of FLH alone or in combination with QS-21. (f) Kinetics of tumor appearance in mice during an experiment similar to that described in (a), designed to analyze the tumor infiltration of immune cells. The percentage of mice bearing tumors was determined visually and by palpation. Quantitative analysis of tumor infiltration based on the numbers of NK1.1^+^ (g), F4/80^+^ (h), CD3^+^ (i), CD4^+^ (j), and CD8^+^ (k) cells in single-cell suspensions derived from the tumors was performed on day 30 of the bioassay. The results are expressed as the percentage of total viable immune cells. The data shown represent means ± SEM of a representative experiment, with *n* = 3 mice per group. ^∗^*P* < 0.05 and ^∗∗^*P* < 0.01, two-tailed Student's *t*-test.

To determine the antitumor effects of FLH in combination with QS-21, we first investigated its effects in a prophylactic setting using a heterotopic oral cancer model with C57Bl/6 mice. KLH was included as a positive control due to its extensive history of safe and effective use in bladder cancer immunotherapy [4, 57]. The scheme of the bioassay is shown in Figure 2(a). The mice previously primed with KLH or FLH alone or in combination with QS-21 were challenged with 1 × 10^5^ MOC7 cells injected into the right flank; after 7 days, the mice underwent intralesional immunotherapy five times at intervals of 3 days. A similar scheme was used with the controls: PBS (vehicle) and QS-21 alone. Figures 2(b) and 2(c) show the progression of tumor volume at 24 days with KLH and FLH treatment, respectively, demonstrating that tumors tended to grow faster in the PBS treatment group than in the mice under immunotherapy with hemocyanins alone or under the suppressive effects of QS-21 alone. However, a survival analysis, which was carried out until day 67 for ethical reasons, showed that 100% of the mice in the PBS and QS-21 control groups died by day 40 and day 55, respectively, and 100% of the mice treated with KLH died by day 45 (Figure 2(d)), in contrast to a 33.3% survival rate for the mice treated with FLH alone and FLH in combination with QS-21 adjuvant (Figure 2(e)).

Another comparable bioassay was performed to explore the infiltration of immune cells in mouse tumors. In this assay, immunotherapy began 24 hours post-challenge with MOC7 cells and continued with similar doses at intervals of 3 days for a total of 5 administrations. These mice were euthanized at 30 days, when all the mice showed tumors (Figure 2(f)). An analysis of infiltrating NK cells showed an increased number with both hemocyanins alone (Figure 2(g)), as previously reported for KLH [28, 61] and FLH [13]; on the contrary, both combinations containing QS-21 showed a decrease in NK cell number. Macrophages showed a significant increase in the PBS group compared to all the other treatments, with the exception of KLH in combination with QS-21 (Figure 2(h)). Notably, FLH treatments alone significantly increased the total level of CD3^+^ among T cells (Figure 2(i)) and significantly decreased the level of CD4^+^ among T cells (Figure 2(j)) compared to an increasing tendency towards CD8^+^ T cells (Figure 2(k)). In contrast, KLH and FLH in combination with QS-21 tended to increase the level of infiltrating CD4^+^ and CD8^+^ T cells. Previous studies investigating the prognostic value of infiltrating lymphocyte profiles in pretreatment specimens from patients with oral cancer have shown a positive correlation between a high number of infiltrating CD3^+^ and CD8^+^ cells and clinical outcome [62]. Therefore, we hypothesized that complementary immunotherapy with FLH in combination with QS-21, which reinforces this profile, would likely be highly beneficial in patients who demonstrate decreased CD3^+^ and CD8^+^ levels prior to the initiation of chemoradiotherapy.

### 3.3. Hemocyanins in Combination with QS-21 Adjuvant Showed Improved Immunotherapeutic Effects in an Orthotopic Model of Oral Cancer

**Figure 3:**
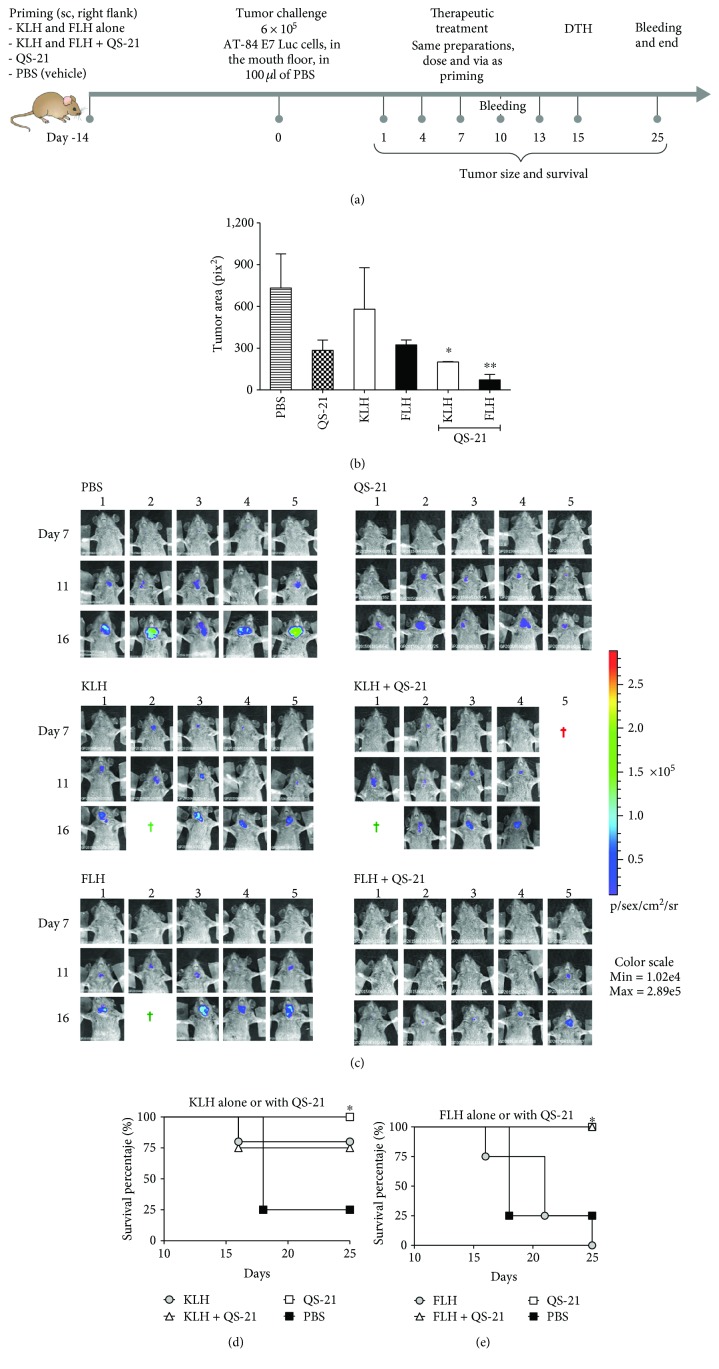
Antitumor effects of KLH and FLH in combination with QS-21 adjuvant in an orthotopic mouse model of oral cancer. (a) Experimental scheme. Five C3H/He mice were primed subcutaneously in the superior right flank (day −14) with the following treatments: 100 *μ*g of hemocyanin alone in 100 *μ*l of PBS (vehicle) or 50 *μ*g of each hemocyanin in combination with 10 *μ*g of QS-21 in a total volume of 100 *μ*l of PBS. The controls were 10 *μ*g of QS-21 alone in 100 *μ*l of PBS and 100 *μ*l of PBS. For the tumor cell challenge (0 days), the mice received 1 × 10^5^ AT-87 Luc cells in the mouth floor. After 24 days, the animals received a subcutaneous injection in the superior right flank identical to the priming dose, which was then repeated 4 times at intervals of 3 days. DTH determination was performed on day 15 of the bioassay. Tumor growth was monitored with the IVIS® Lumina imaging system and palpation. For acquisition, the mice were anesthetized and injected intraperitoneally with 150 mg/kg D-luciferin; 10 minutes later, the animals were exposed to the CCD camera for 5 minutes. (b) Tumor growth at day 16. The tumor areas of the mice receiving PBS treatment were compared to those of the mice treated with hemocyanins alone and in combination with QS-21 as well with the adjuvant alone. Data are shown as means ± SEM of one representative experiment with *n* = 5 mice per group for FLH + QS-21 and QS-21, four mice per group for KLH and FLH, and three mice per group for KLH + QS-21. ^∗^*P* < 0.05 and ^∗∗^*P* < 0.01, two-tailed Student's *t*-test. (c) Representative murine bioluminescence images. Representative *in vivo* images (IVIS) of luminescence shown in the mouth floor of live C3H/He mice on days 7, 11, and 16 after the start of the experiment. Images obtained from ventral views of the mice show relative levels of luminescence ranging from low (blue) to medium (green) to high (yellow/red); the scale on the right indicates the average radiance. The green crosses represent animals that were euthanized, and the red crosses represent animals that died after the application of anesthesia. (d) Survival analysis of mice. A Kaplan-Meier survival analysis was performed using the log-rank test. ^∗^*P* < 0.05 for one representative experiment with *n* = 5 mice per group, with the exception of the FLH plus QS-21 group (four mice).

We next evaluated FLH and KLH preparations in combination with QS-21 in a prophylactic setting in an oral cancer model, which is more predictive of the clinical outcome of therapeutic vaccines for this cancer than the above nonorthotopic model [31]. The scheme of this bioassay is shown in Figure 3(a). The mice previously primed with FLH or KLH alone or in combination with QS-21, as well as PBS and QS-21 alone as controls, were challenged with 6 × 10^5^ bioluminescent AT-84 E7 Luc cells injected into the mouth floor of C3H mice via an extraoral route to generate orthotopic tumors. The mice then underwent systemic therapy via subcutaneous injection into their right flanks 24 hours post-challenge with tumor cells, and similar doses were applied 4 times at 3-day intervals. As shown in Figure 3(b), on day 16, one day after the final therapy administration, the group of mice treated with KLH and FLH in combination with QS-21 demonstrated the lowest tumor volume when compared with the controls and with the hemocyanin-only treatments. In addition, to track the invasion of the tumors in the mouth floors of the mice, images were recorded *in vivo* with an imaging system (IVIS).  Figure 3(c) shows images taken at 7, 11, and 16 days for all the mice under experimentation. The color scale is shown: blue indicates lower luminescence (zones with less tumor tissue), and red indicates higher luminescence (zones with more tumor tissue). Moreover, the individual causes of death as the trial progressed are indicated: during anesthesia (red crosses) or by euthanasia due to a tumor volume of approximately 2,000 mm^3^ (green crosses). The mice in the PBS control group demonstrated the highest amounts of tumor tissue compared with the mice that received the FLH and KLH treatments in combination with QS-21. Figures 3(d) and 3(e) summarize the survival of the mice receiving KLH and FLH treatments, respectively, until 25 days (excluding mice that died during anesthesia or were euthanized), showing that 75% of mice treated with KLH alone or in combination with QS-21 survived, and 100% of the mice treated with QS-21 or FLH in combination with QS-21 reached the end of the experiment (Figure 3(e)).

**Figure 4:**
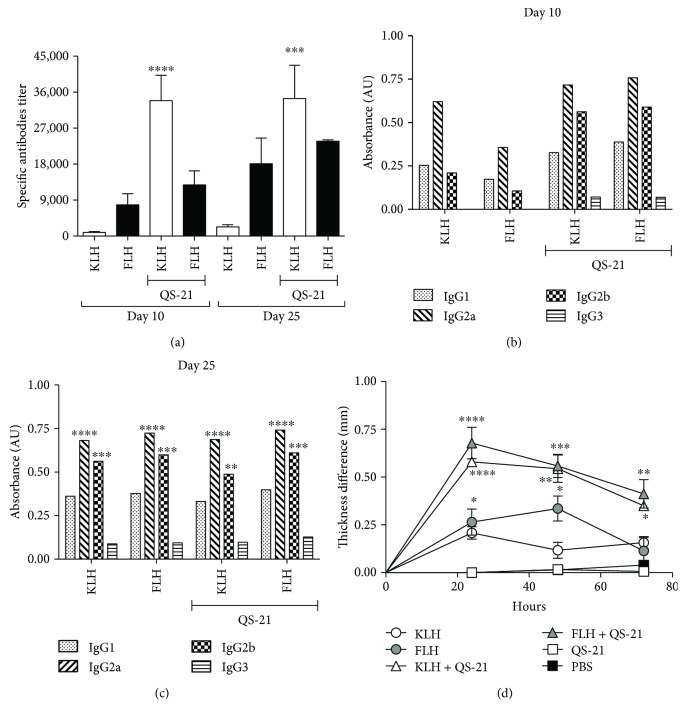
Humoral and cellular immune responses of the mice from the prophylactic bioassay utilizing the oral cancer orthotopic model consisting of C3H/He mice challenged with the AT-84 E7 Luc cell line. Groups of five female C3H/He mice were immunized with one of the following treatments: 100 *μ*g of KLH or FLH, 50 *μ*g of KLH or FLH plus 10 *μ*g of QS-21, and 10 *μ*g of QS-21 or 100 *μ*l of PBS. At 10 and 25 days, serum samples were taken to determine the titers of IgG subclasses by indirect ELISA. DTH determination was performed on day 15 of the bioassay. (a) Total anti-hemocyanin IgG titers in mouse sera. Data are shown as means ± SEM. Anti-hemocyanin IgG titers for each hemocyanin alone were compared by one-way ANOVA with the same hemocyanin in combination with QS-21 at day 10 and at day 21. ^∗∗∗∗^*P* < 0.0001 and ^∗∗∗^*P* < 0.001. Representative experiment with *n* = 5 mice per group. (b) Anti-hemocyanin IgG subclass antibodies at day 10. Half of the maximum OD obtained for each of the isotypes with the different treatments was used for comparison [28]. (c) Anti-hemocyanin IgG subclass antibodies at day 25. Half of the maximum OD obtained for each of the isotypes with the different treatments was used for comparison [28]. IgG1 was compared against IgG2a, IgG2b, and IgG3 for each treatment. ^∗∗∗∗^*P* < 0.0001, ^∗∗∗^*P* < 0.001, and ^∗∗^*P* < 0.01, two-way ANOVA. (d) Delayed hypersensitivity reaction. This assay was performed by applying the corresponding hemocyanin for each treatment group to the left paw of the mouse and subsequently measuring the difference in thickness from the right paw after 24, 48, and 72 hours. Data are shown as means ± SEM of a representative experiment with *n* = 5 mice per group. The foot thickness in the control group was compared at different measurement time points with those in the other treatments at the same time points. ^∗∗∗∗^*P* < 0.0001, ^∗∗∗^*P* < 0.001, ^∗∗^*P* < 0.01, and ^∗^*P* < 0.05, analyzed by one-way ANOVA.

When evaluating the anti-hemocyanin IgG titers in the mouse sera at 10 and 25 days, the highest titers were obtained with hemocyanins in combination with QS-21 compared with hemocyanins alone, as expected (Figure 4(a)). At 10 days, IgG subclass analyses showed a balanced Th1/Th2 type response without significant differences between IgG1 and IgG2a and IgG2b and IgG3 (Figure 4(b)). In contrast, at 25 days, a characteristic Th1-type immune response, characterized by an increase in IgG2a and IgG2b with respect to IgG1, was observed for hemocyanins both alone and in combination with QS-21 (Figure 1(c)), confirming the results obtained with C57BL/6 mice. Notably, these analyses were focused on IgG-type antibodies because they fulfill pivotal effector functions in cancer immunotherapy, including antibody-dependent cytotoxicity and complement activation by the classical pathway; these functions are supported by the chimeric and humanized IgG isotype antibodies against various tumor antigens that are used today for the treatment of different cancers [63]. Moreover, we recently demonstrated that CCH, FLH, and KLH elicited complement activation mediated by C1 binding to human natural antibodies that cross-react with these glycoproteins [64]. Finally, there was a significant difference in DTH testing with respect to the controls (PBS and QS-21) at all time points analyzed. KLH or FLH alone or both hemocyanins in combination with QS-21 increased DTH responses, with the highest significant values scored by the latter formulations (Figure 4(d)). Our data are similar to those previously reported by Kim et al., in which QS-21 induced DTH reactivity for KLH as a carrier of a tumor-associated antigen [65].

Taken together, the results obtained in this study allow us to highlight the future possibilities offered by this original new mouse model of oral cancer, which mimics human oral cancer produced by HPV, and to further monitor the growth of tumor cells implanted in the mouth floor with high sensitivity and precision because AT-84 E7 Luc cells incorporate the E7 protein genes from HPV and the luciferase enzyme [31]. In addition, hemocyanin formulations may have beneficial applications for the immunotherapy of cancers as aggressive as oral cancer. Notably, these early experiments have inherent limitations, because it is necessary to select an immunotherapeutic treatment scheme that limits the range of analyses. Therefore, additional time course and dose-response experiments are required.

### 3.4. QS-21 Adjuvant Exerts Cytotoxic Effects *In Vitro* on MOC7 and AT-84 E7 Luc Oral Cancer Cell Lines

**Figure 5:**
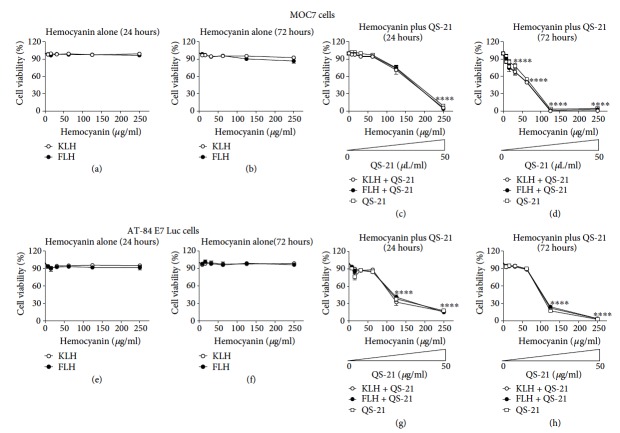
Effects of hemocyanins in combination with QS-21 on the viability of the MOC7 and AT-84 E7 Luc oral cancer cell lines. Cells (6 × 10^5^ cells/well) were incubated with different concentrations of hemocyanin (KLH or FLH) and QS-21 for 24 and 72 hours, and viability was determined using the alamarBlue® method. (a) Effects on MOC7 cell viability with KLH or FLH after 24 hours of culture. (b) Effects on MOC7 cell viability with KLH or FLH after 72 hours of culture. (c) Effects on MOC7 cell viability with KLH or FLH in combination with QS-21 after 24 hours of culture. The concentration gradient of this adjuvant, described in Methods, is indicated. (d) Effects on MOC7 cell viability with KLH or FLH in combination with QS-21 after 72 hours of culture. (e) Effects on AT-87 Luc cell viability with KLH or FLH after 24 hours of culture. (f) Effects on AT-87 Luc cell viability with KLH or FLH after 72 hours of culture. (g) Effects on AT-87 Luc cell viability with KLH or FLH in combination with QS-21 after 24 hours of culture. The concentration gradient of this adjuvant, described in Methods, is indicated. (h) Effects on MOC7 cell viability with KLH or FLH in combination with QS-21 after 72 hours of culture. In each case, the cell viability values obtained at different formulation concentrations were compared with those obtained for the corresponding untreated condition. ^∗∗∗∗^*P* < 0.0001, one-way ANOVA; *n* = 2 independent experiments performed in triplicate.

Due to the antitumor effects observed with the QS-21 adjuvant alone in the bioassays described previously, we analyzed the effects of QS-21 both alone and in combination with hemocyanins on the *in vitro* proliferation of the MOC7 and AT-84 E7 Luc cell lines. Different concentrations of KLH and FLH were evaluated, starting from a concentration close to that utilized in the experimental animals. In the case of hemocyanin in combination with QS-21, the adjuvant concentrations used were proportional to those of hemocyanin, using as a reference the concentration at which each of these substances was applied to prime the mice. Cell viability was compared with the value obtained for a similar culture without treatment after 24 and 72 hours of incubation.  Figure 5 shows that neither KLH nor FLH affected the viability of either oral cancer cell line at the different concentrations and times tested (Figures 5(a) and 5(b) for MOC7 cells, 5(e) and 5(f) for AT-84-E7 Luc cells). However, the cells cultured with QS-21 showed cytotoxic effects both alone and with hemocyanins in both oral cancer cell lines (Figures 5(c) and 5(d) for MOC7 cells, 5(g) and 5(h) for AT-84 E7 LUC cells).

**Figure 6:**
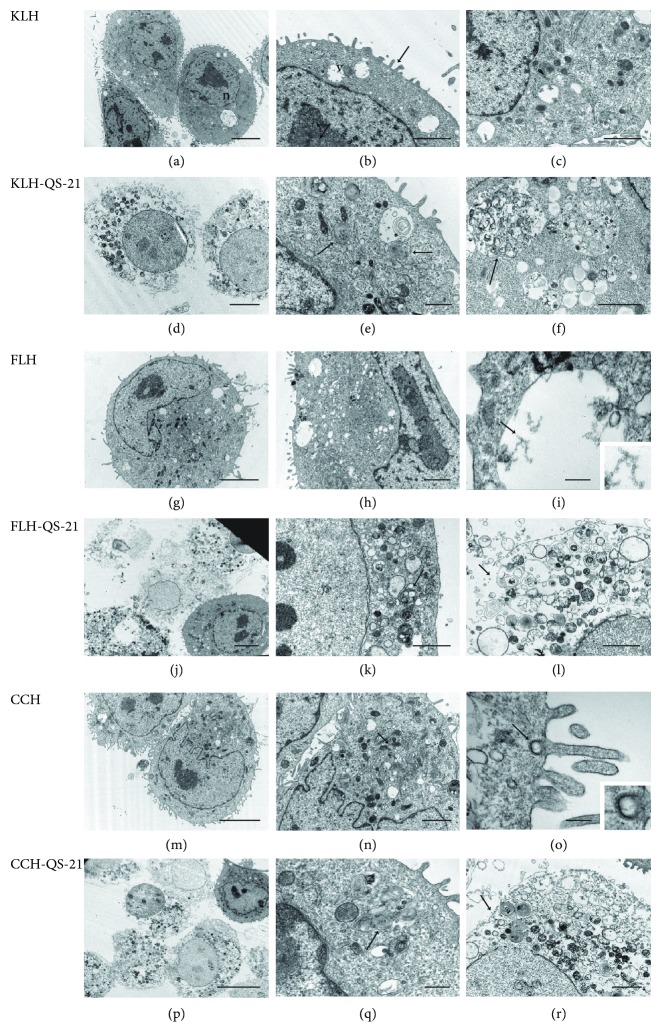
Ultrastructural TEM analysis of MOC7 cells cultured with hemocyanins combined with QS-21. Transmission electron micrographs of MOC cells after 24 hours of incubation with hemocyanins alone or in combination with QS-21. The culture conditions are specified in Methods. (a) Cells incubated with KLH. Low-magnification image of the cells showing their normal appearance, i.e., an eccentric nucleus (n) and abundant cytoplasmic organelles. Scale bar represents 5 *μ*m. (b) Details of the cytoplasm showing small, clear vacuoles (v); the surface of the cells with extended prolongations (arrow). Scale bar represents 2 *μ*m. (c) Details of the cytoplasm. Scale bar represents 2 *μ*m. (d) Cells incubated with KLH in combination with QS-21. Low-magnification image of the cells showing degenerative changes. Scale bar represents 2 *μ*m. (e) Cytoplasm exhibiting vacuoles with multimembrane bodies. Scale bar represents 1 *μ*m. (f) Details of vacuoles showing membranous and particulate contents. Scale bar represents 2 *μ*m. (g) Cells incubated with FLH. Low-magnification image of a cell showing its normal appearance. Scale bar represents 5 *μ*m. (h) Details of the cytoplasm. Scale bar represents 0.5 *μ*m. (i) FLH molecules inside clear vacuoles. A higher-magnification inset image of hemocyanin molecules in the area is indicated with an arrow. Scale bar represents 0.2 *μ*m. (j) Cells incubated with KLH in combination with QS-21. Scale bar represents 5 *μ*m. (k) Low-magnification image of cells showing their cytoplasm with several multimembrane bodies. Scale bar represents 2 *μ*m. (l) Details of a cytolyzed cell. Scale bar represents 2 *μ*m. (m) Cells incubated with CCH. Low-magnification image of cells showing their normal appearance. Scale bar represents 5 *μ*m. (n) Details of the cytoplasm showing profuse organelles. Scale bar represents 2 *μ*m. (o) A coated vesicle containing CCH molecules. Higher-magnification inset image of hemocyanin molecules in the area, indicated with an arrow. Scale bar represents 0.2 *μ*m. (p) Cells incubated with CCH in combination with QS-21. Low-magnification image of the cells showing their abnormal appearance. Scale bar represents 10 *μ*m. (q) Cytoplasm showing vacuoles with multimembrane bodies. Scale bar represents 0.5 *μ*m. (r) Details of a cytolyzed cell with a ruptured plasma membrane (arrow). Scale bar represents 2 *μ*m.

These results were supported by an ultrastructural TEM analysis in MOC7 cells to investigate the toxic effects described above at the morphological level. As shown in Figure 6, the cells cultured with KLH, FLH, and CCH alone showed normal morphology characterized by an intact cell membrane, an eccentric nucleus, and profuse cytoplasmic organelles (Figures 6(a)to 6(c) for KLH, 6(g)to 6(i) for FLH, and 6(m)to 6(o) for CCH). Moreover, hemocyanin molecules were found in clear vacuoles in the cytoplasm of the cells, indicative of micropinocytosis (Figure 6(i)) and clathrin-mediated endocytosis (Figure 6(o)). In contrast, in MOC7 cells cultured with hemocyanins in combination with QS-21 adjuvant, most cells showed evident degeneration, which was characterized by rupture of the plasma membrane, provoking cell lysis, and the presence of multimembrane structures in the cytoplasm, characteristic of apoptotic and autophagic-appearing structures [66, 67] (Figures 6(d)to 6(f) for KLH, 6(j)to 6(l) for FLH, and 6(p)to 6(r) for CCH).

## 4. Discussion

The use of KLH in biomedicine has a long history, highlighted by its nonspecific immunomodulatory effects to prevent recurrence after transurethral surgical resection of superficial bladder cancer [4, 5]. However, the use of KLH, as well as that of the newer CCH and FLH, in the context of other cancers has been poorly documented, most likely because its nonspecific immunomodulatory effects are not sufficient against more aggressive and deadly malignant cancers such as oral squamous cell carcinoma. During the development of HNSCC, frequent mutations occur, which would create neoantigens, indicating that immunotherapies might be an effective therapeutic strategy [68]. Therefore, to further boost the adaptive immune response induced by these hemocyanins, in the present study, different adjuvants that have been shown to be safe and effective in human vaccines were evaluated: alum, AddaVax, and QS-21. Of these three adjuvants, QS-21 combined with hemocyanins showed the best results, as reflected by a robust, specific humoral response predominantly characterized by IgG2a antibodies and a sustained cellular response manifesting as a DTH reaction, confirming that QS-21 stimulates the polarization of the cytokine environment to Th1 response; although only half the recommended dose of QS-21 was used in the mice, AddaVax and alum were used at the maximum recommended dose [25, 69].

Comparing the hemocyanins used here, FLH showed intrinsically greater immunogenic capabilities, a result consistent with those of our previous studies demonstrating the superior performance of immunogens based on FLH, which is partially explained by its activation of a more potent innate immune response. In fact, FLH can rapidly induce the secretion of certain proinflammatory cytokines by antigen-presenting cells, including IL-6, TNF-*α*, IL-12p40, and IL-23*α*, a phenomenon that may explain its enhanced immunological activities [13]. Moreover, we recently demonstrated that CCH and FLH, similar to KLH, are safe and useful carriers of mimotopes of tumor-associated carbohydrate antigens, such as the P10 mimotope of GD2 ganglioside, the major ganglioside constituent of neuroectodermal tumors, and that preparations based on FLH-P10, similar to KLH-P10, showed antitumor effects superior to those of CCH-P10 in a melanoma model [25].

The humoral immune responses observed with KLH and QS-21 are consistent with those in previous reports using KLH covalently coupled to tumor-associated antigens of melanoma [39]. Indeed, when the effects of several immunological adjuvants combined with GD3 ganglioside and MUC1 peptide coupled to KLH were compared, QS-21 was the most effective at inducing specific IgM and IgG antibodies against both MUC1 and GD3 [39]. Furthermore, positive results achieved by combining KLH and QS-21 compared to other adjuvants were also reported in the development of T-cell [70] and B-cell lymphoma vaccines [71]. Indeed, a formulation consisting of a human CD20 peptide coupled to KLH and combined with QS-21 produced higher specific titers of anti-CD20 serum antibodies than a formulation using alum [71]. Notably, however, no previous study in a murine cancer model has described the nonspecific immunomodulatory effects of a formulation consisting of one mollusk hemocyanin in combination with QS-21, as presented in the current study.

Using a heterotopic model of oral cancer and a novel orthotopic model that uses bioluminescent oral cancer cells to monitor tumor progression *in vivo*, we demonstrated here that KLH and FLH in combination with QS-21 could reduce tumor development and increase mouse survival. Notably, in this orthotopic C3H/He murine bioassay, treatment application was systemic (by subcutaneous inoculation in the right flank) and not intralesional, as in our previous studies investigating the immunomodulatory effects of hemocyanins [13, 25, 28, 72]. Herein, for the first time, we showed that hemocyanin formulations alone or in combination with QS-21 were able to induce positive antitumor activity when applied at a site distant from a tumor. However, despite positive results, several animals from the groups treated with QS-21 showed skin damage, demonstrating QS-21 toxicity [73, 74]. In humans, the adverse effects of this saponin limit the dosage to 50 *μ*g, except in the case of cancer patients [75]. Therefore, it will be necessary in future experiments to use lower doses of QS-21 than those used in this study to reduce side effects in the experimental animals. This adjuvant has been proposed to induce localized, controlled cell death; thus, the “danger signals” released from injured cells contribute to its potent immunologic activity [76]. It is important to mention that the most beneficial effects of hemocyanins were obtained after priming with these glycoproteins before tumor cell challenge; however, in human beings affected by oral cancer, such pretreatment is not possible, and patients must be treated after disease initiation. Nevertheless, some studies have shown a higher incidence of oral cancer in certain human population groups [77, 78], and the use of a vaccine of this nature in these populations could be considered.

For most anticancer agents, the initial step in their evaluation as therapeutic agents is the determination of any effects on *in vitro* cell cultures, as these models are less expensive and less time-consuming than experiments with *in vivo* animal tumor models [79]. Therefore, to detect the possible mechanism of action of the hemocyanin plus QS-21 adjuvant preparations, their effects on oral cancer cell lines viability were evaluated *in vitro*. The results demonstrated that neither KLH nor FLH affected the viability of oral cancer cell lines at the tested concentrations and times. Although previous studies have evaluated the effects of KLH on different tumor cell lines cultured *in vitro* [8–11], the data provided in these prior works cannot be compared with our results. In some cases, the amount of hemocyanin added to the cultures was indicated, but its concentration was not, whereas in other cases, the amount of KLH per well but not the total volume was indicated, as is the case with melanoma lines [10, 11]. Thus, it was impossible to extrapolate the actual concentration of protein used. The only work that completely describes the KLH concentration is that of Riggs et al., which used breast and pancreatic cancer lines [9]. These authors reported a significant decrease in cell viability and increase in apoptotic cell death at 500 and 250 *μ*g/ml 72 hours after hemocyanin administration. In contrast, our results with 250 *μ*/ml KLH did not show evidence of a cytotoxic effect. We did not test the concentration of 500 *μ*g/ml because this dose would never be achieved with the treatments applied in the performed bioassays with mice. There is no prior information for FLH or CCH or for these two hemocyanins in combination with QS-21. However, there is evidence that other hemocyanins obtained from *Helix aspersa* [80] and *Rapana venosa* [81] have cytotoxic and antiproliferative effects on tumor cell lines such as bladder cancer, ovarian cancer, prostate cancer, and glioma cancer cell lines.

Our data suggest that the *in vivo* effects of KLH and FLH in oral carcinoma are associated with their modulation of the immune response rather than with cytotoxic effects on tissue cells at the site of inoculation. The precise mechanisms underlying the nonspecific immunostimulatory capacities of these hemocyanins in some cancers remain to be elucidated. We previously demonstrated that hemocyanins are incorporated by antigen-presenting cells, both by micropinocytosis and by clathrin-mediated endocytosis, and these cells become activated while slowly processing these very large glycoproteins [26]. As a consequence, hemocyanins induce an inflammatory milieu promoting the secretion of Th1 cytokines [82]. Although a DTH test with extracts of the oral cell lines was not performed in the animals used for the antitumor bioassays, a preliminary experiment showed that, for animals under immunotherapy with FLH plus QS-21, challenge with an extract of MOC7 cells induced a DTH-positive reaction (data not shown), suggesting that this formulation encourages a specific cellular immune response against tumor cells. Therefore, we hypothesized that these glycoproteins lead to the stimulation of CD8^+^ T lymphocytes against several tumor-associated antigens, either by the cross-reaction of anti-hemocyanin glycotope antibodies with carbohydrates localized to tumor cell surfaces or by the indirect stimulation of latent specific immune responses (the bystander effect), impairing the immune tolerance of the tumor [25]. A biomedical application of this knowledge is the design of therapeutic cancer vaccines through the generation of ex vivo autologous DCs loaded with tumor lysates for the induction of polyclonal T-cell expansion [83]. Remarkably, the efficiency of this type of vaccination procedure is increased when the cells are also loaded with KLH [84] or CCH [29] as adjuvants. Thus, hemocyanin contributes to the reversal of the tolerogenic profile of DCs from cancer patients towards an immunostimulatory profile [85] characterized by a bias towards a Th1-type immune response.

## 5. Conclusions

Our data demonstrated that hemocyanins in combination with QS-21 induced robust humoral and cell-mediated immune responses compared to hemocyanins in combination with alum or AddaVax. Using a heterotopic and a novel orthotopic model of oral cancer, we demonstrated that KLH and FLH in combination with QS-21 can affect tumor growth. In addition, we reported that KLH and FLH, unlike QS-21, have no cytotoxic effect on oral cancer cells, supporting the idea that the antitumor effects of hemocyanins are associated with their modulation of the immune response. Hemocyanin utilization would allow reduction of the QS-21 dosage, which is effective but causes toxic as well as therapeutic results. Overall, our exploratory study highlights the potential applications of hemocyanins combined with an adjuvant in the development of immunotherapeutic strategies for oral squamous cell carcinoma.

## Data Availability

The datasets generated during and/or analyzed during the current study are available from the corresponding author on reasonable request.

## Abbreviations

CCH: *Concholepas concholepas* hemocyanin
DC: Dendritic cell
DTH: Delayed-type hypersensitivity
FBS: Fetal bovine serum
FLH: *Fissurella latimarginata* hemocyanin
HNSCC: Head and neck squamous cell carcinoma
HPV: Human papilloma virus
IVIS: In vivo imagin system
KLH: Keyhole limpet hemocyanin
PBS: Phosphate-buffered saline
ROI: Region of interest
SBC: Superficial bladder cancer
TEM: Transmission electron microscopy.
